# Digital Cover Photography for Estimating Leaf Area Index (LAI) in Apple Trees Using a Variable Light Extinction Coefficient

**DOI:** 10.3390/s150202860

**Published:** 2015-01-28

**Authors:** Carlos Poblete-Echeverría, Sigfredo Fuentes, Samuel Ortega-Farias, Jaime Gonzalez-Talice, Jose Antonio Yuri

**Affiliations:** 1 Research and Extension Center for Irrigation and Agroclimatology (CITRA), Universidad de Talca, Talca 3460000, Chile; E-Mails: sortega@utalca.cl; 2 Faculty of Veterinary and Agricultural Sciences, University of Melbourne, Melbourne, VIC 3010, Australia; E-Mail: sigfredo.fuentes@unimelb.edu.au; 3 Centro de Pomaceas, Universidad de Talca, Talca 3460000, Chile; E-Mails: jgonzalezt@utalca.cl (J.G.-T.); ayuri@utalca.cl (J.A.Y.)

**Keywords:** light intercepted by the canopy, gap fraction, clumping index, remote sensing

## Abstract

Leaf area index (LAI) is one of the key biophysical variables required for crop modeling. Direct LAI measurements are time consuming and difficult to obtain for experimental and commercial fruit orchards. Devices used to estimate LAI have shown considerable errors when compared to ground-truth or destructive measurements, requiring tedious site-specific calibrations. The objective of this study was to test the performance of a modified digital cover photography method to estimate LAI in apple trees using conventional digital photography and instantaneous measurements of incident radiation (I_o_) and transmitted radiation (I) through the canopy. Leaf area of 40 single apple trees were measured destructively to obtain real leaf area index (LAI_D_), which was compared with LAI estimated by the proposed digital photography method (LAI_M_). Results showed that the LAI_M_ was able to estimate LAI_D_ with an error of 25% using a constant light extinction coefficient (k = 0.68). However, when k was estimated using an exponential function based on the fraction of foliage cover (f_f_) derived from images, the error was reduced to 18%. Furthermore, when measurements of light intercepted by the canopy (I_c_) were used as a proxy value for k, the method presented an error of only 9%. These results have shown that by using a proxy k value, estimated by I_c_, helped to increase accuracy of LAI estimates using digital cover images for apple trees with different canopy sizes and under field conditions.

## 1. Introduction

Accurate modeling of energy balance, gas exchange processes and light distribution occurring within the canopy of fruit orchards requires the characterization and assessment of canopy vigor and structure. Leaf area index (LAI) is one of the most important variables to assess canopy structure [1]. Moreover, LAI is a key variable used by a variety of physiological and functional plant models [2] and by remote sensing models at large scales [3–5].

Direct measurements of LAI in field conditions are extremely difficult to obtain for experimental and commercial fruit orchards. The usual technique to determine LAI is based on the analysis of destructive samples by collecting leaves and the subsequent measurement of their area using leaf area meters (e.g., Li-3100C; Li-Cor, Lincoln, NE, USA), or by digitally acquiring leaf scans and image processing. These methods are destructive, labor intensive, and can be significantly expensive when applied to large trees [6]. In general, these methods have been used to develop empirical allometric equations based on measurements of total leaf area relative to shoot length or plant area related to trunk diameter. Allometric equations are widely used to estimate LAI for different crops and to validate other indirect LAI estimation methods [7]. However, these equations are site-specific and may vary with changes in canopy size and climatic conditions [8,9].

Alternatively, indirect optical methods have been developed based on measurements of direct or diffuse light penetration through the canopy [10–13]. Different devices have been developed to estimate LAI using light transmittance measurements, such as: (I) the plant canopy analyzer (PCA) LAI-2000 (or 2200) (Licor Inc., Lincoln, NE, USA), which uses a fisheye light sensor that measures diffuse radiation simultaneously in five distinct angles [6,10,14]; (II) the DEMON (Centre for Environmental Mechanics, Canberra, Australia), which is based on measurements of direct radiation to estimate LAI [10,13]; (III) SunScan (Delta-T devices Ltd. Cambridge, UK) and AccuPAR (Decagon Devices, Pullman, WA, USA), which measures photosynthetic active radiation (PAR) with wavelengths between 400–700 nm [10]; (IV) hemispherical and fisheye photography to assess canopy structure through image analysis. The problem with the latter imagery techniques is that they require a complex image analysis procedure and specific software [2,10,15,16]. The optical methods to assess LAI have been successfully tested for different crops. However, the direct application of these techniques requires the use of destructive measurements to calibrate the instruments for each specific condition, species and location since they usually underestimate LAI compared to direct measurements [10,16–18].

Due to the practical constraints of current methodologies available to estimate LAI, the development of a simple, accurate and practical method is required to assess the necessary parameters to estimate LAI, particularly under field conditions for experimental purposes. Recently, accurate and rapid estimations of LAI have been made possible through the development of a simple method that uses cover digital photography and gap fraction analysis [2,16–18]. The main objective of this study was to analyze the performance of a modified automated procedure, proposed by Fuentes *et al.* [2,19]. This new procedure was developed to estimate LAI in apple orchards using a proxy light extinction coefficient (k) obtained from digital photography and instantaneous measurements of incident (I_o_) and transmitted radiation below the canopy (I) [20].

## 2. Materials and Methods

### 2.1. Experimental Sites Description

The study was conducted in the 2009–2010 agricultural season in two commercial apple orchards. The first trial (Trial 1) was carried out in an orchard planted in 2007 with apple trees cv. Cripp's Pink/M-7, located in the Maule Region, Pelarco, Chile (35°25′L.S; 71°23′L.W., 189 m.a.s.l.). The planting distance was 3 m (between rows) × 1.5 m (between plants) (2,222 trees·ha^−1^) in East-West oriented rows with a Solaxe training system. The experimental plot had 10 trees with heights ranging from 2.5 to 3.0 m. The second trial (Trial 2) was carried out with apple trees cv. Ultra Red Gala/MM 111, planted in 2003 and located in the Maule Region, San Clemente (35°30′L.S; 71°28′L.W., 83 m.a.s.l.). The planting distance was 4 m (between rows) × 2 m (between plants) (1250 trees·ha^−1^) in East-West oriented rows with the Solaxe training system [21]. In Trial 2 the experimental plot had 12 trees with heights ranging from 3.8 to 4.0 m (cv. Ultra Red Gala_1_) and 18 trees with heights ranging from 2.5 to 3.0 m (cv. Ultra Red Gala_2_). For both experimental sites, the topping was performed just above a lateral productive branch. The climate in both orchards is a Mediterranean type with a mean maximum temperature of 30 °C in the warmest month (January) and a mean minimum temperature of 3.5 °C in the coldest month (July). The mean annual rainfall is 700 mm, with a dry period of six months (November to April). In this study digital cover photography acquisition and canopy light interception measurements were carried out in February and April for apple trees cv. Ultra Red Gala and cv. Cripp's Pink, respectively.

### 2.2. Destructive Estimation of Leaf Area Index (LAI)

Leaf area (LA) per plant was assessed for the 40 trees used in this study (10 trees cv. Cripp's Pink, 18 trees cv. Ultra Red Gala_1_ (3.8–4.0 m tall) and 12 trees cv. Ultra Red Gala_2_ (2.5–3.0 m tall)). After taking digital images and light measurements from tree canopies, plants were completely defoliated. Leaves were stored in cooler containers and immediately taken to the laboratory for analysis. A sub-sample of 200 g of fresh leaves per tree was taken and their areas were measured with a leaf area meter (LI-3100 Area meter, Li-Cor Biosciences, Lincoln, NE, USA.). This procedure allowed measurement of the specific leaf area (SLA) expressed in cm^2^·g^−1^. Total tree leaf area was obtained by multiplying the total leaf mass from the whole tree by the SLA obtained. Then, destructive leaf area index (LAI_D_) was calculated by dividing total tree leaf area by the space assigned per tree (distance between rows multiplied by distance between plants, assuming equal size trees). The size of the sub-sample used in this study was validated in a previous study of apple trees in the same orchards through comparison between LAI_D_ estimated by the subsample and LAI_D_ obtained by measurements of total leaves of trees [22]. Furthermore, since fruits and branches also contribute to the light extinction through the canopy, digital image acquisition and light canopy interception measurements were carried out in the presence of fruits on the trees for comparative purposes between trials.

### 2.3. Digital Cover Photography Acquisition

**Figure 1.**
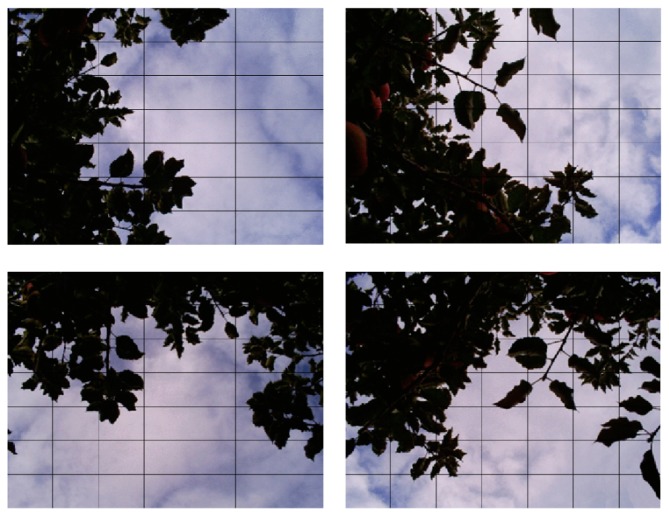
Example of typical upward looking digital images for an apple tree, considering the four quadrants defined with an image sub-division of 7 (49 sub-samples per each image).

A conventional digital camera (Digimax A503, Samsung, Korea) with focal length of 36 mm and angles of view of 53.13° horizontal, 36.87° vertical and 62° diagonal (field of view (FOV) at 1 m distance equal to 0.964 m wide × 0.643 m high) was mounted on a tripod with a bubble level to ensure the camera was in the horizontal position for each image. Digital images were acquired with a resolution of 2048 × 1536 pixels in the Joint Photographic Experts Group (JPEG) format at the zenith angle from all plants as described by Fuentes *et al.* [2]. Digital images were collected with automatic exposure at 0.3 m from the ground 4 h before noon to avoid direct sunlight shinning into the lens of the camera. Apple trees were divided into four quadrants and four images were obtained per tree (Figure 1), resulting in a total of 160 digital images (40 trees).

### 2.4. Canopy Light Interception Measurements

Incident radiation (I_o_) and the radiation transmitted below canopy (I) were measured using a commercial Ceptometer (AccuPAR LP-80, Decagon Devices Inc., Pullman, WA, USA), by taking one measurement above the canopy and five measurements distributed below the canopy parallel to tree rows. The measurements were regularly spaced between two rows every 0.3 m for the Trial 1 and 0.4 m for the Trial 2 on both sides of the tree trunks. Ceptometer measurements were taken in parallel to digital images (8 February 8th in cv. Ultra Red Gala and 25 April 25 in cv. Cripp's Pink). The below canopy readings were averaged to calculate the light intercepted by the canopy (I_c_) as follows: I_c_ = 1 − I/I_o_ (dimensionless).

### 2.5. Leaf Area Index Estimated by the Digital Photography Method (LAI_M_)

The analysis script developed by Fuentes *et al.* [2] performs a cloud filtering process and automatic gap analysis of upward-looking digital images. The cloud filtering process is applied by analyzing image color and brightness. The blue band (450–495 nm) is used to filter clouds, since they provide the best contrast between foliage cover and sky plus clouds [2]. The blue band of each image was extracted as a histogram and explored to identify a suitable threshold between foliage and sky. The code developed allows the automation of this process by identifying the minima value automatically between the peaks from sky and foliage.

The automatic gap analysis is performed by dividing each binary image into a number of sub-images defined by the user. For this study, we used 4 images per tree with an image sub-division of 7 (49 sub-images per digital image, 196 sub-images per tree) as standard parameters (Figure 1). From each sub-image, the program counts the total of pixels corresponding to sky (S) and leaves (L). A large gap is considered when the ratio S/L in each sub-image is larger than a user-specified value. In this study, a large gap threshold equal to 0.75 was used. This value was proposed by Fuentes *et al.* [6] for evergreen Eucalyptus woodland. In addition, in the same study, it was shown that changes in the threshold presented a low contribution in the final LAI estimation assessed through sensitivity analysis. When this threshold criterion is met per sub-image, the pixel count for S is added to the big gap count for that particular full image. If the ratio observed is smaller than the user-specified value for a specific sub-image, the pixel count contribution to the total big gap count of that particular sub-image is equal to zero. On the other hand, the light extinction coefficient (k) can be incorporated as a fixed value or as a variable value per image. The fractions of foliage cover (f_f_) and crown cover (f_c_) are calculated from Mcfarlane *et al.* [16] using the following equations:

(1) $${\text{f}}_{\text{f}}=1-\frac{{\text{g}}_{\text{T}}}{{\text{T}}_{\text{P}}}$$

(2) $${\text{f}}_{\text{c}}=1-\frac{{\text{g}}_{\text{L}}}{{\text{T}}_{\text{P}}}$$

where g_T_ is the total number of gap pixels; g_L_ is the total number of large gap pixels and T_P_ is the total number of pixels. Using the f_f_ and f_c_ calculated values, the crown porosity (Φ) can be calculated as follows:

(3) $$\Phi =1-\frac{{\text{f}}_{\text{f}}}{{\text{f}}_{\text{c}}}$$

The clumping index at the zenith (Ω(0)) and the effective leaf area index (LAI_M_) are calculated from Beer's Law as follows [2,16,23]:

(4) $$\Omega (0)=\frac{\left(1-\Phi )\cdot \ln(1-{\text{f}}_{\text{f}}\right)}{\ln(\Phi )\cdot {\text{f}}_{\text{f}}}$$

(5) $${\text{LAI}}_{\text{M}}=-{\text{f}}_{\text{c}}\cdot \left(\frac{\ln(\Phi )}{\text{k}}\right)\cdot \Omega (0)$$

Finally, the measured light extinction coefficient (k_M_) was calculated by inverting Equation (5) using the measured value of LAI (LAI_D_) as follows:

(6) $${\text{k}}_{\text{M}}=-{\text{f}}_{\text{c}}\cdot \left(\frac{\ln(\Phi )}{{\text{LAI}}_{\text{D}}}\right)\cdot \Omega (0)$$

### 2.6. Performance Evaluation of the Digital Photography Method (LAI_M_)

The evaluation of the performance of LAI_M_ included a linear regression between LAI_D_ and LAI_M_, the calculation of root mean square error (RMSE), mean absolute error (MAE), mean bias error (MBE) and index of agreement (d) [24–26]. Additionally, a sensitivity analysis was carried out to evaluate the effect of variations (of ±30%) in k in the estimation of LAI_M_. Furthermore, the relationships between k_M_ *versus* f_c_ and k_M_ *versus* I_c_ were evaluated by linear and exponential models.

## 3. Results

The averaged values obtained for LAI_D_, k_M_, f_f_, f_c_, Φ and Ω(0), for the cv. Cripp's Pink (Trial 1) and cv. Ultra Red Gala_1_ and cv. Ultra Red Gala_2_ (Trial 2) are summarized in Table 1. When comparing both experimental sites, the cv. Cripp's Pink presented the lowest values of LAI_D_ and SLA with average values of 1.84 and 29.9 (cm^2^·g^−1^), respectively. Similar results were observed considering the rest of the parameters analyzed, with the exception of crown porosity (Φ), which was the highest. The cv. Ultra Red Gala_2_ presented the highest LAI_D_ = 2.96 and correspondingly lowest Φ = 0.14. Tallest trees (cv. Ultra Red Gala_1_, 3.8–4.0 m tall) presented the highest k_M_ with an average value of 0.79. Additionally, the cv. Ultra Red Gala_1&2_ presented Ω(0) values closest to 1. The variability within trees was not significant, as presented by the standard deviation values (SD) for the different parameters. The sensitivity analysis of k_M_ (used to estimate LAI_M_) showed that LAI_M_ was significantly affected by ±30% variation of k_M_. The relative LAI_M_ changes generated by variations of k_M_ + 30% and k_M_ − 30% were in the order of −23.1% and 42.9%, respectively.

### 3.1. Estimation of Light Extinction Coefficient

**Figure 2.**
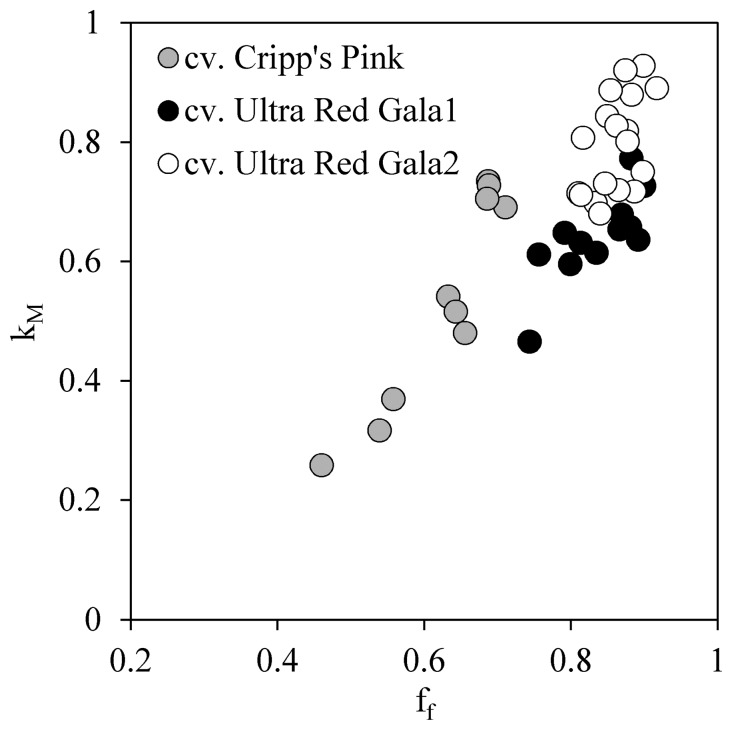
Exponential relationship between measured light extinction coefficient (k_M_) and fraction of foliage cover (f_f_). Dashed line represents the exponential model for the whole dataset, the continuous line represents the exponential model for the Trial 1 (cv. Cripp's Pink) and dotted line represents the exponential model for the Trial 2 (cv. Ultra Red Gala_1_ and cv. Ultra Red Gala_2_).

An exponential relationship was found when comparing the light extinction coefficient estimated by the fractions of foliage cover (f_f_) *versus* the k_M_ obtained by inverting Equation (5). Considering the whole dataset the r^2^ found for the exponential model was 0.67. A similar fit was obtained using a linear model with an r^2^ equal to 0.62. (Table 2). Figure 2 shows that the exponential relationships obtained are site and cultivar specific. In the case of cv. Cripp's Pink, the exponential model showed an r^2^ equal to 0.93, while for cv. Ultra Red Gala_1&2_ the r^2^ value for the exponential model was equal to 0.46 (Table 2).

**Figure 3.**
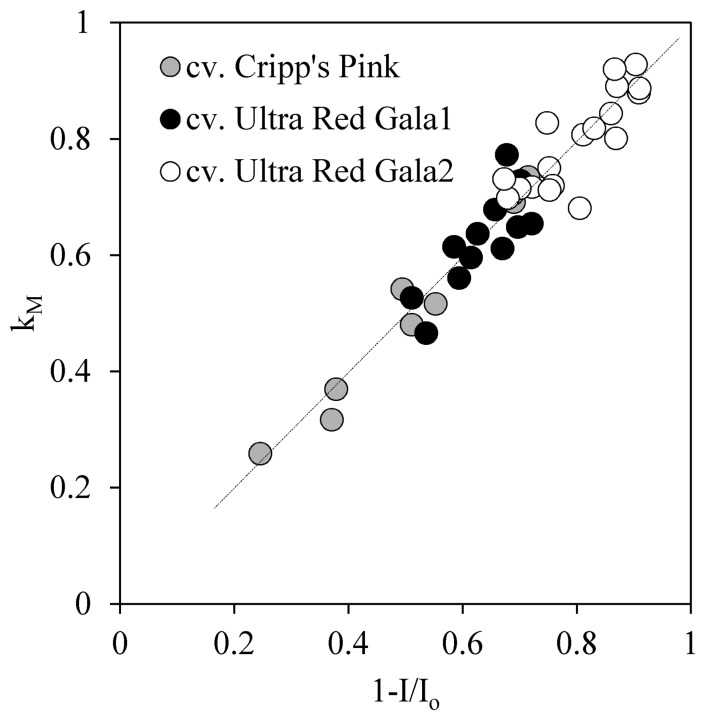
Relationship between measured light extinction coefficient (k_M_) and the fraction of light intercepted by the canopy (I_c_ = 1 − I/I_o_) for both trials.

Furthermore, we tested the use of instantaneous canopy light interception measurements as a proxy for k_M_ (Figure 3). When the whole dataset was considered (*n* = 40), the instantaneous fraction of light intercepted by the canopy (I_c_) showed a linear relationship with k_M_. The linear regression analysis (forced to pass through the origin) between k_M_ and I_c_ was highly significant (*p* < 0.01) with an r^2^ value of 0.90 and slope *b* = 0.99. Figure 3 shows that the data cloud was closely distributed to the 1:1 line with a range of variability of k_M_ *vs.* I_c_ from a minimum of 0.25 to a maximum of 0.95.

### 3.2. Determination of LAI_M_

The LAI_M_ was calculated using three approaches: (I) using parameters derived from the image analysis and a constant light extinction coefficient (k = 0.68) (LAI_M1_); (II) using parameters derived from the image analysis with k_M_ estimated by an exponential function of f_f_ (LAI_M2_) and (III) using f_c_, f_f_, Φ and Ω(0) derived from the image analysis and the instantaneous values of the fraction of incident radiation absorbed by the canopy (LAI_M3_).

The comparison between LAI_D_ *vs.* LAI_M1_, LAI_M2_ and LAI_M3_ is presented in Table 3. This table shows that LAI_M1_ had RMSE of 0.61, equivalent to 25% of error. In this case the method was tested using a common extinction value, k = 0.68, for all images (*n* = 160), and resulted in a poor correlation between the LAI_D_ and LAI_M1_ (r^2^ = 0.3). LAI_M2_ had a RMSE of 0.44 (18% of error) and r^2^ = 0.40. Finally, the LAI_M3_ displayed a much better agreement with LAI_D_, with a d value of 0.96. Furthermore, the values of RMSE and MAE were 0.22 (9% of error) and 0.17 (7% of error), respectively.

**Figure 4.**
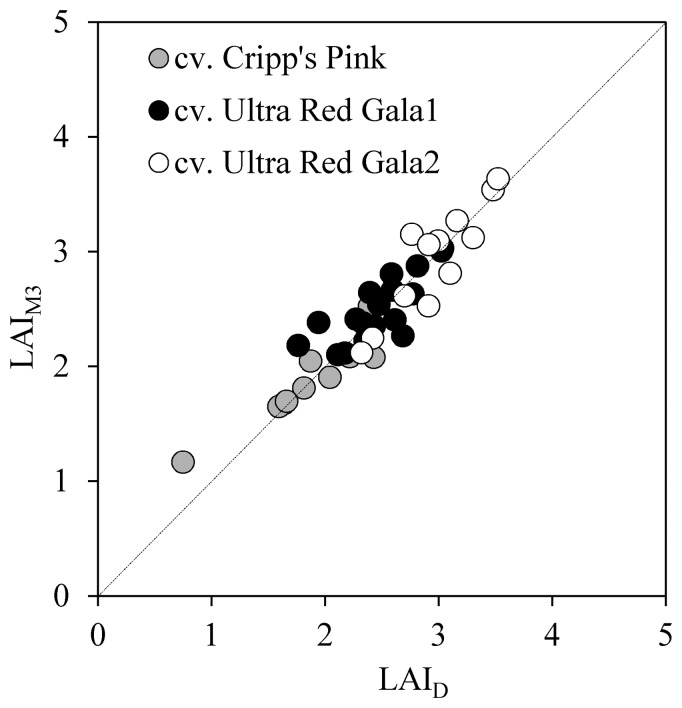
Comparison between leaf area index estimated by cover digital images considering instantaneous fraction of incident radiation absorbed by the canopy (LAI_M3_) *vs.* leaf area index obtained by defoliated method (LAI_D_). Data shown considers all varieties and study fields.

Linear regression analysis between LAI_D_ and LAI_M3_ for the whole dataset (40 apple trees) was highly significant with an r^2^ value of 0.85 (Figure 4). The t-tests for the null hypothesis of intercept = 0 and slope = 1.0 at the 95.0% confidence level were accepted with *p*-values of 0.55 and 0.58, respectively. Figure 4 shows the linear relationship between LAI_D_ and LAI_M3_ for the three measurement sites. The same figure shows a data point that escaped from the main data cloud, corresponding to a tree from the cv. Cripp's Pink variety that presented LAI_D_ values of around 1.0. The range of LAI_D_ obtained from the data varied from 1.0 to a maximum of 3.75.

## 4. Discussions

In this study, measurements of canopy light interception and digital cover images were taken four hours before midday with the objective of avoiding direct sunlight within the pictures. This consideration for timing consistency in imaging is very important as in fruit tree orchards radiation transmittance varies greatly throughout the day [27–30]. Additionally, Zhao *et al.* [30] showed that there is significant influence of daytime on the pixel count between vegetation and background in vertical upward-oriented digital images. Their main recommendation was to avoid images taken under direct light conditions in order to ensure that the sensed radiation does not include any reflected or transmitted radiation by leaves.

Previous studies have proposed the use of an automated procedure using cover photography with a single averaged k per tree species. Specifically for eucalyptus, a k = 0.5 has been recommended [2,16]. In the case of grapevines, a calculated k = 0.7 has also been reported [31] and used as a constant k [32]. However, in the present study, it was demonstrated that the cover photography method (LAI_M_) is sensitive to k values required to obtain accurate LAI from isolated apple trees. Therefore the accuracy of the method depends on a good estimator of k for individual images. According to results presented here, using a constant k for apples (k = 0.68 average value obtained from Table 1) will result in an overestimation of the averaged k for Cripp's Pink of 20% and an underestimation for the averaged k for Ultra Red Gala_1_ of 16% and 7.4% for Ultra Red Gala_2_. Furthermore, it was shown that an over and underestimation of 30% from k_M_ resulted in overestimations and underestimations of LAI_D_ by 43% and 23%, respectively, which are significant differences from real values. The differences in k obtained per apple cultivar and per trial site highlight how crucial local calibration for any LAI methodology can be. This can be considered the main constraint for indirect measurement methods that deal with light transmission through the canopy. Another constraint in using other common LAI measurement methodologies is that local calibrations are generally useful only for those same conditions of canopy vigor and site. Therefore, local calibrations cannot be used in other cultivars or same cultivars with different canopy structure (Table 3). Differences between both experimental sites could be related mainly to the age of the orchards. In Trial 2 the three-year old plants had not reached their productive potential and had yet not occupied the total assigned ground area. Therefore a higher proportion of leaf area growth will be more vertically elongated towards areas with large gaps and areas with low porosity than in more balanced trees (Trial 1), where growth is characterized by a more uniform leaf area distribution.

The automated methodology presented in this paper can incorporate an independent k value per image analyzed. This can be obtained either using a parameter measured from the same image, such as f_f_ or parallel measurements of the fraction of light intercepted by the canopy (I_c_). Macfarlane *et al.* [17] showed that f_f_ was highly correlated with LAI obtained using allometry and the plant canopy analyzer (LI-2000) in *E. globulus* stands (r^2^ values of 0.78 and 0.82, respectively). In this study by using a proxy k, obtained from f_f_ (LAI_M2_), the model explained only 40% of the LAI_D_ variability with an associated RMSE of 18%. A positive aspect of this method is that it is completely automated, since the analysis code is able to calculate k using the equation found in LAI_M1_. The downside of the model is that it requires the calibration of f_f_ against k_M_ to obtain the algorithm for other cultivars and species, since the relationship between f_f_ and k_M_ depends on the characteristic of the orchard, specifically the plant density. Results in Figure 2 show that the relationship between f_f_ and k_M_ is site and cultivar specific. When a specific model is used for Trial 1 and 2 the RMSE in the LAI_M2_ method is reduced to 15%.

Most significantly, by using a proxy k value obtained by the relationship between k_M_ and I_c_ = 1 − I/I_o_, which explained 85% of the variability of k_M_, the LAI_M3_ estimation improved significantly with an associated RMSE of only 9%, This could be attributed to the inclusion of fruits and non-leaf material, such as branches in the images. The positive aspect of this method is that the I_c_ factor can be a direct input in the estimation of k value required per image to calculate LAI_M_. This relationship was also consistent for both cultivars unlike the relation found with f_f_. The downside of this latter method is that it requires the measurement of I and I_o_ per image using another instrument. To aid in this process a light sensor is currently under development for smartphone and tablet platform use, allowing easy assessment of light interception above and below the canopy [32]. Furthermore, the I_c_ factor takes into consideration the specific canopy architecture and vigor of trees. Therefore, this procedure can be considered as an easy auto-calibration procedure per image, which offers more accurate estimations of LAI per image while considering site specificity effectively.

## 5. Conclusions

The demonstrated, automated methodology, for estimating LAI from apple trees using digital cover photography and variable light extinction coefficient has been shown to be an accurate and inexpensive technique to estimate LAI and other architectural parameters. Results have shown that the LAI_M_ method was able to estimate LAI_D_ with an error of 18% when the light extinction coefficient (k) was simulated as a function of fraction of foliage cover (f_f_) derived from digital images. However, when variable measurements of light intercepted by the canopy (I_c_) were used as a proxy for k, light extinction, digital photography method presented an RMSE of only 9%. These results have demonstrated that by using a variable k, estimated by I_c_, the accuracy of LAI estimates for apple trees with variable canopy sizes and under field conditions increased significantly. This method can be used for experimental and practical applications. The latter could allow growers to identify spatial differences in vigor that could be correlated to unseen factors, such as soil differences. Plant functional models and remote sensing techniques could benefit from the proposed methodology as a consequence of simplifying the acquisition and analysis of accurate LAI.

## Figures and Tables

**Table 1.** Average and standard deviation values obtained for destructive leaf area index (LAI_D_), specific leaf area (SLA), light intercepted by the canopy (I_c_), measured extinction coefficient (k_M_), fraction of foliage cover (f_f_), crown cover (f_c_), crown porosity (Φ) and clumping index at the zenith angle (Ω(0)) for the both trial sites.

**Trial**		**LAI_D_**	**SLA**	**I_c_**	**k_M_**	**f_f_**	**f_c_**	**Φ**	**Ω(0)**
cv. Cripp's Pink (*n* = 10, Trial 1)	* Avg.	1.84	28.0	0.551	0.553	0.66	0.76	0.17	0.75
	** S.D.	0.49	2.92	0.165	0.179	0.11	0.09	0.04	0.07

cv. Ultra Red Gala_1_ (*n* = 18, Trail 2)	* Avg.	2.46	28.2	0.795	0.793	0.86	0.98	0.12	0.97
	** S.D.	0.34	1.25	0.083	0.087	0.03	0.01	0.02	0.02

cv. Ultra Red Gala_2_ (*n* = 12, Trail 2)	* Avg.	2.96	34.1	0.632	0.625	0.83	0.97	0.14	0.96
	** S.D.	0.38	1.16	0.067	0.084	0.05	0.03	0.04	0.04

Total (*n* = 40)	* Avg.	2.46	29.9	0.683	0.680	0.80	0.92	0.14	0.91
	** S.D.	0.57	3.54	0.145	0.152	0.11	0.11	0.04	0.10

* Avg. is the average value;

** S.D. is the standard deviation; SLA expressed in cm^2^·g^−1^.

**Table 2.** Models for light extinction coefficient as a function of the fractions of foliage cover (f_f_) for the both trial sites.

**Trial**	**Model Type**	**Equation**	**r^2^**
Trial 1 (cv. Cripp's Pink)	Exponential	k_M_ = 0.031·exp (4.44·f_f_)	0.93
	Linear	k_M_ = 2.02·f_f_ − 0.73	0.87

Trial 2 (cv. Ultra Red Gala_1&2_)	Exponential	k_M_ = 0.096·exp (2.37·f_f_).	0.46
	Linear	k_M_ = 1.63·f_f_ − 0.65	0.42

Whole dataset	Exponential	k_M_ = 0.136·exp (1.99·f_f_).	0.67
	Linear	k_M_ = 1.08·f_f_ − 0.17	0.62

r^2^ is the coefficient of determination (dimensionless).

**Table 3.** Statistical analysis of leaf area index estimated by the LAI_M_ method using a constant k value (LAI_M1_), a k derived from f_f_ (LAI_M2_) and using the instantaneous fraction of incident radiation absorbed by the canopy as a proxy value for k (LAI_M3_).

**Approach**	**RMSE**	**MAE**	**MBE**	**r^2^**	**d**
LAI_M1_	0.61 (25%)	0.46 (19%)	0.07 (3%)	0.30	0.70
LAI_M2_	0.44 (18%)	0.36 (15%)	−0.06 (−2.4%)	0.40	0.71
LAI_M3_	0.22 (9%)	0.17(7%)	0.01 (0.2%)	0.85	0.96

RMSE is the root mean square error (mm·day^−1^); MBE is the mean bias error (mm·day^−1^); MAE is the mean absolute error (mm·day^−1^); r^2^ is the coefficient of determination (dimensionless); d is the index of agreement (dimensionless).
